# Mineralocorticoid Receptor Antagonists Mitigate Mitral Regurgitation-Induced Myocardial Dysfunction

**DOI:** 10.3390/cells11172750

**Published:** 2022-09-03

**Authors:** Wei-Ting Chang, Yu-Wen Lin, Chin-Yu Chen, Zhih-Cherng Chen, Jhih-Yuan Shih, Chia-Ching Wu, Chwan-Yau Luo, Ping-Yen Liu

**Affiliations:** 1 Institute of Clinical Medicine, College of Medicine, National Cheng Kung University, Tainan 70101, Taiwan; 2 Division of Cardiology, Department of Internal Medicine, Chi-Mei Medical Center, Tainan 71004, Taiwan; 3 Department of Biotechnology, Southern Taiwan University of Science and Technology, Tainan 71004, Taiwan; 4 Department of Radiology, Chi-Mei Medical Center, Tainan 71004, Taiwan; 5 Department of Health and Nutrition, Chia Nan University of Pharmacy and Science, Tainan 71710, Taiwan; 6 Department of Cell Biology and Anatomy, College of Medicine, National Cheng Kung University, Tainan 70101, Taiwan; 7 Institute of Basic Medical Sciences, College of Medicine, National Cheng Kung University, Tainan 70101, Taiwan; 8 International Center for Wound Repair and Regeneration, National Cheng Kung University, Tainan 70101, Taiwan; 9 Division of Cardiovascular Surgery, Department of Surgery, National Cheng Kung University Hospital, College of Medicine, National Cheng Kung University, Tainan 70101, Taiwan; 10 Division of Cardiovascular Surgery, Department of Surgery, Kaohsiung Medical University, Kaohsiung 807378, Taiwan; 11 Division of Cardiology, Department of Internal Medicine, National Cheng Kung University Hospital, College of Medicine, National Cheng Kung University, Tainan 70101, Taiwan

**Keywords:** mitral regurgitation, heart failure, cardiac fibrosis, ER stress, apoptosis

## Abstract

Mitral regurgitation (MR), the disruption of the mitral valve, contributes to heart failure (HF). Under conditions of volume overload, excess mineralocorticoids promote cardiac fibrosis. The mineralocorticoid receptor antagonist spironolactone is a potassium-sparing diuretic and a guideline-recommended therapy for HF, but whether it can ameliorate degenerative MR remains unknown. Herein, we investigate the efficacy of spironolactone in improving cardiac remodeling in MR-induced HF compared with that of a loop diuretic, furosemide. Using a novel and mini-invasive technique, we established a rat model of MR. We treated the rats with spironolactone or furosemide for twelve weeks. The levels of cardiac fibrosis, apoptosis, and stress-associated proteins were then measured. In parallel, we compared the cardiac remodeling of 165 patients with degenerative MR receiving either spironolactone or furosemide. Echocardiography was performed at baseline and at six months. In MR rats treated with spironolactone, left ventricular function—especially when strained—and the pressure volume relationship significantly improved compared to those of rats treated with furosemide. Spironolactone treatment demonstrated significant attenuation of cardiac fibrosis and apoptosis in left ventricular tissue compared to furosemide. Further, spironolactone suppressed the expression of apoptosis-, NADPH oxidase 4 (NOX4)- and inducible nitric oxide synthase (iNOS)-associated proteins. Similarly, compared with MR patients receiving furosemide those prescribed spironolactone demonstrated a trend toward reduction in MR severity and showed improvement in left ventricular function. Collectively, MR-induced cardiovascular dysfunction, including fibrosis and apoptosis, was effectively attenuated by spironolactone treatment. Our findings suggest a potential therapeutic option for degenerative MR-induced HF.

## 1. Introduction

With an aging society, valvular heart disease (VHD) is a major contributor to heart failure (HF) [1]. Mitral regurgitation (MR), which is common, consists of degenerative (primary) and functional (secondary) abnormalities [1]. In contrast to functional MR involving myocardial dysfunction that secondarily affects valve performance, degenerative MR involves intrinsic structural abnormalities in valves [1,2]. In some cases, functional MR, cased by the chamber dilatation and non-coaptation, could be improved once myocardial function improves. Conversely, to date, effective pharmacological therapy for degenerative MR, caused by the damage of valve structure per se, is lacking, while surgical interventions can only arrest the pathophysiologic process [1,3].

The mineralocorticoid pathway is crucial for activating cardiac fibroblasts and endothelial cells, resulting in cardiac fibrosis and remodeling [4]. Spironolactone (Spiro), an antagonist of mineralocorticoid receptors, has been found to reduce myocardial fibrosis and endothelial–mesenchymal transition in mouse models of chronic aortic regurgitation and mitral valve prolapse [4,5,6]. Clinically, the Randomized Aldactone Evaluation Study (RALES) reported that Spiro use was associated with a 30% and 35% reduction, respectively, in mortality and rehospitalization in severe HF [7]. The beneficial effect was thought to be attributed to the ability of Spiro to mitigate myocardial fibrosis. However, whether Spiro can also suppress degenerative MR-induced myocardial dysfunction remains largely unknown. In our study, both clinical observation and a novel mini-invasive rat model of degenerative MR [8] were used to investigate the efficacy of Spiro in mitigating the severity of MR and induced myocardial fibrosis compared with another loop diuretic, furosemide (Furo).

## 2. Materials and Methods

### 2.1. Animals and Experimental Design

10-week-old adult male Sprague Dawley rats (250–300 g) were obtained from the Animal Center of Bengbu Medical College (Bengbu, China). All animal procedures were approved by the Research Animal Care of Chi-Mei Medical Center and conformed to the Guide for the Care and Use of Laboratory Animals. For MR model, using a 22-gauge needle we performed a perforating defect at the leaflet under the echocardiography guidance [8]. After surgery, Sprague Dawley rats (n = 48) were randomly divided into the following four groups: sham, MR (untreated), MR+Spiro (100 mg/kg/day), and MR+Furo (30 mg/kg/day). Drugs were given once daily by gastric gavage for 12 weeks according to previous literature [9]. Systolic blood pressure was measured every 2 weeks by the indirect tail-cuff method after MR creation. The detailed experimental design is shown in Supplemental Figure S1.

### 2.2. Echocardiographic and Strain Analyses in Animals

Echocardiography was performed on anesthetized rats using GE Vivid S6 Dimension echocardiography platform with a 10 MHz linear array transducer (GE-Vingmed Ultrasound AS, Horten, Norway) every 2 weeks after MR creation. The details of echocardiography in rats are displayed in Supplemental Methods.

### 2.3. Hemodynamic Study of Pressure-Volume Loop (PV Loop)

At the end of the experiment, the hemodynamic changes in the left ventricle (LV) were measured by pressure–volume (P-V) loop as previously described [10]. The details of hemodynamics in rats were measured by PV loop and are addressed in Supplemental Methods.

### 2.4. Histological Analysis

At the end of the study, heart specimens were obtained from the ventricular septum and immediately fixed in 4% formalin, then embedded in paraffin. The specimens were serially cut at 4 µm and deparaffinized with a graded series of xylene and ethanol solutions. Cardiac histology was measured by hematoxylin-eosin (H&E) staining. The fibrosis of LV was assessed by Masson’s trichrome staining and analyzed using Image J software. In situ DNA fragmentation was performed to detect cardiac apoptosis in LV using terminal deoxynucleotidyl transferase-mediated dUTP nick end labeling assay kit (TUNEL; BioVision, Milpitas, CA, USA) according to the manufacturer’s protocol. Nuclei were counterstained with 4′, 6-diamidino-2-phenylindole (DAPI, Vector Laboratories, Newark, CA). TUNEL-positive cells were examined under a fluorescence microscope (Olympus BX51, Olympus Optical Co. Ltd., Tokyo, Japan). In addition, 8-Hydroxydeoxyguanosine (8OHdG; Bioss Antibodies, Woburn, MA, USA) staining was applied to detect the tissue expressions of reactive oxygen species (ROS). Those results are presented as the percentage of positive to total cells.

### 2.5. Western Blot

Protein expressions in LV tissues of rats were measured by Western blot. The details of methods and antibodies information are displayed in Supplemental Methods.

### 2.6. Patients and Clinical Study Designs

In this retrospective study, we collected the clinical and echocardiographic parameters of patients diagnosed with degenerative MR of at least moderate severity at Chi-Mei Medical Center (enrollment period 2017 to 2019). The degree of MR in the study was defined by the recommended guidelines of the American Society of Echocardiography for effective regurgitant orifice area (EROA) ≥ 0.4 cm^2^ and regurgitation volume ≥ 60 mL lasting >6 months [11]. Patients prescribed either Spiro of Furo for at least three consecutive months were included, while those who took both Spiro and Furo were excluded. Other exclusion criteria included symptomatic hypotension, a history of hyperkalemia, decompensated HF, acute coronary syndrome (ACS), stroke, cardiovascular surgery, or percutaneous coronary intervention within six months. All other drugs, including angiotensin-converting enzyme inhibitors/angiotensin receptor blockers (ACEIs/ARBs), β-blockers, statins, antiarrhythmic drugs and antiplatelet/anticoagulants, were continued. Echocardiographic parameters, including changes in MR severity and left ventricular structure and functions, were assessed before and six months after medications (Spiro of Furo). The study was conducted in strict accordance with the Declaration of Helsinki on Biomedical Research involving human subjects and was approved by the local ethics committee (IRB: 10307-003).

### 2.7. Echocardiographic Parameters in Patients

Transthoracic echocardiography (GE E9, Vingmed Ultrasound AS, Horten, Norway) was performed by experienced technicians and confirmed by cardiologists. The details of echocardiographic measurements are displayed in Supplemental Methods.

### 2.8. Statistics

All of the experiments were performed at least three times. Continuous data are presented as mean ± standard deviation or median (interquartile range), depending on the distribution. Dichotomous data are presented as numbers and percentages. Chi-squared test or Fisher’s exact test were used to compare categorical variables as appropriate. Statistical differences among groups were determined using by two-way and one-way ANOVA with Tukey’s multiple comparisons test. Values of *p* < 0.05 were considered to be significant. All analyses were performed using SPSS, version 18 for Windows (SPSS Inc., Chicago, IL, USA). Experimental data of animals were analyzed by GraphPad Prism 6.01 software (La Jolla, CA, USA).

## 3. Results

### 3.1. Spiro Improved Myocardial Strain in a Rat Model of MR

**Figure 1:**
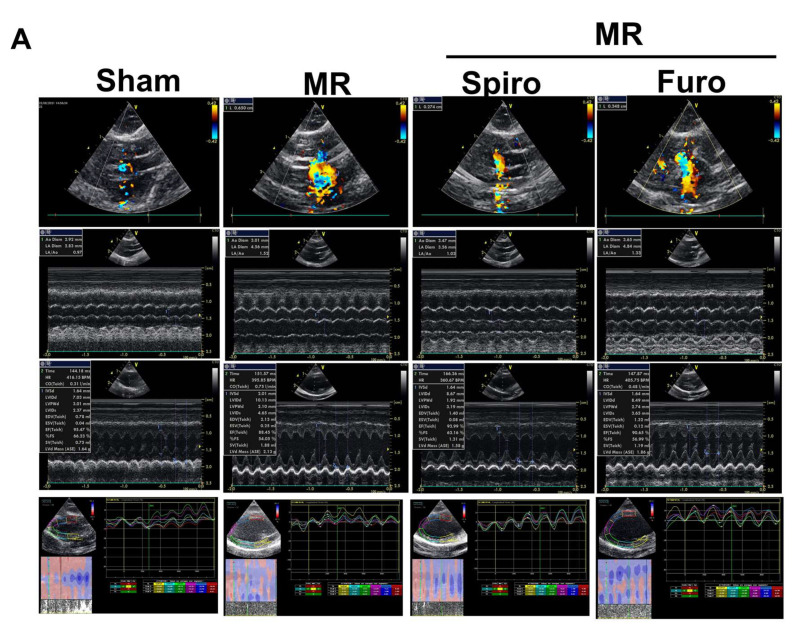

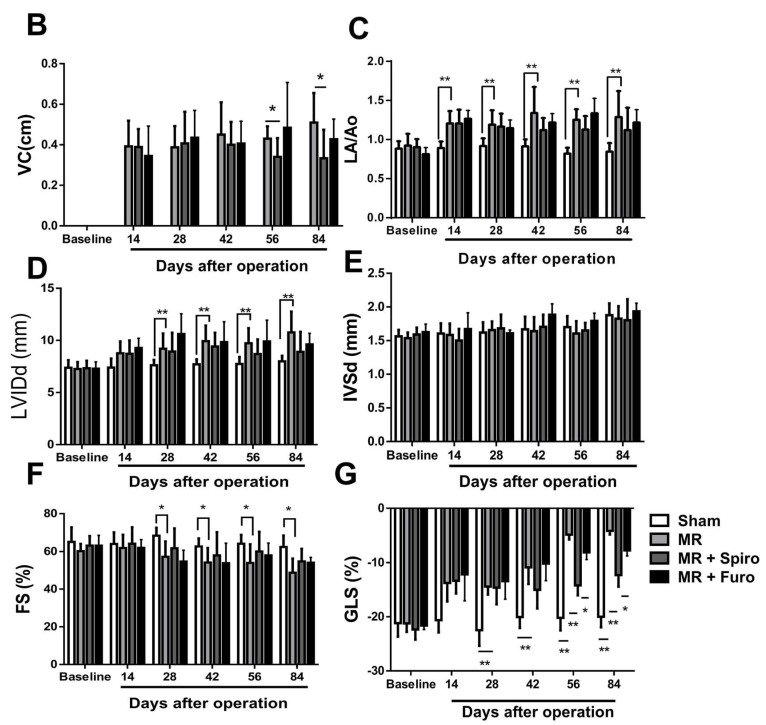
Effect of mineralocorticoid receptor antagonists on the progression of mitral regurgitation (MR) and myocardial dysfunction in a rat model of MR. Heart structure and function were measured by echocardiography at baseline, Day 14 postoperation, Day 28, Day 42, Day 56, and Day 84 post-treatment. (**A**) Illustrations of echocardiographic imaging in the sham, MR, MR+ spironolactone (Spiro), and MR+ furosemide (Furo) groups. (**B**) Echocardiographic measurement results for vena contracta (VC), (**C**) left atrial (LA)/aorta (Ao) size ratio, (**D**) left ventricular internal dimension at end-diastole (LVIDd), (**E**) interventricular septal thickness at end-diastole (IVSd), (**F**) fractional shortening (FS), and (**G**) global longitudinal strain (GLS). Data are expressed as the mean ± standard deviation (S.D.). * *p* < 0.05, ** *p* < 0.01 for differences from each group.

To mimic MR-induced volume overload, we established a novel and mini-invasive MR model in SD rats [8]. Two weeks after the surgery, rats were randomized and treated with Spiro or Furo for 4 consecutive weeks, and cardiac function in the sham, MR, MR+Spiro, and MR+Furo groups was assessed by echocardiography every week (Supplemental Figure S1A). During the twelve-week treatment, there were no significant changes in body weight, heart rate, or blood pressure among the groups (Supplemental Figure S1B–F). As shown in Figure 1A, despite a reduction in MR severity in rats treated with Spiro compared with those treated with Furo (Figure 1B), chamber dimensions did not significantly change after MR was induced (Figure 1C–E). Despite a significant decline in FS in rats post-MR surgery, FS was similar in MR rats treated with Spiro and Furo (Figure 1F). Further, using strain imaging, we found that post-MR induction, global longitudinal strain (GLS) significantly dropped. After 84 days of Spiro treatment, the strain was significantly restored, but Furo was not (Figure 1G).

### 3.2. Spiro Improves MR-Induced Volume Overload in Hemodynamic Studies

**Figure 2:**
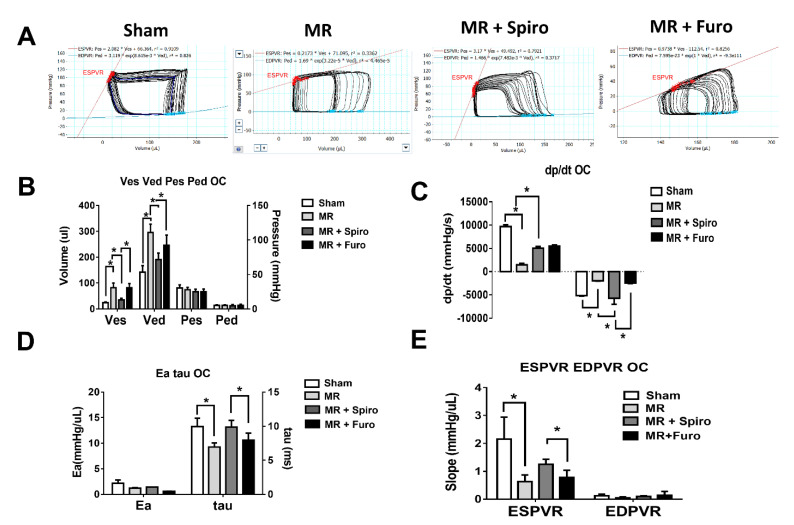
Effects of mineralocorticoid receptor antagonists on cardiac hemodynamics in a rat model of mitral regurgitation (MR). (**A**) Representative pressure–volume plots in the sham, MR, MR+Spiro, and MR+Furo groups. (**B**) Comparison of the mean end-systolic volume (Ves), end-diastolic volume (Ved), end-systolic pressure (Pes), and end-diastolic pressure (Ped); (**C**) maximal velocity of pressure rise (+dP/dt) and fall (−dP/dt); (**D**) mean arterial elastance (Ea), the time constant of isovolumic pressure decay (tau); and (**E**) mean slopes of the end-systolic pressure–volume relationship (ESPVR) and end-diastolic pressure–volume relationship (EDPVR) in each group. * *p* < 0.05 for differences from each group.

Using a pressure–volume loop, we measured hemodynamic parameters in rats twelve weeks postoperatively. Representative LV loop images in the sham, MR, MR+Spiro, and MR+Furo groups are shown in Figure 2A. The MR group had significantly higher left ventricular volumes in both end-systolic and end-diastolic volume (Ves and Ved) than the sham group, indicating that a volume overload was induced by MR surgery. In rats treated with Spiro, a volume overload was significantly improved but was absent in those treated with Furo (Figure 2B). Similarly, R-induced suppression in maximal velocity of pressure rise (+dP/dt) and fall (−dP/dt) was significantly mitigated by Spiro treatment (Figure 2C). Additionally, there was a significant improvement in isovolumic pressure decay (tau) (Figure 2D) and end-systolic pressure–volume relationship (ESPVR) slope in the MR rats treated with Spiro compared with those treated with Furo (Figure 2E).

### 3.3. Spiro Reduced MR-Induced Myocardial Fibrosis and Apoptosis

**Figure 3:**
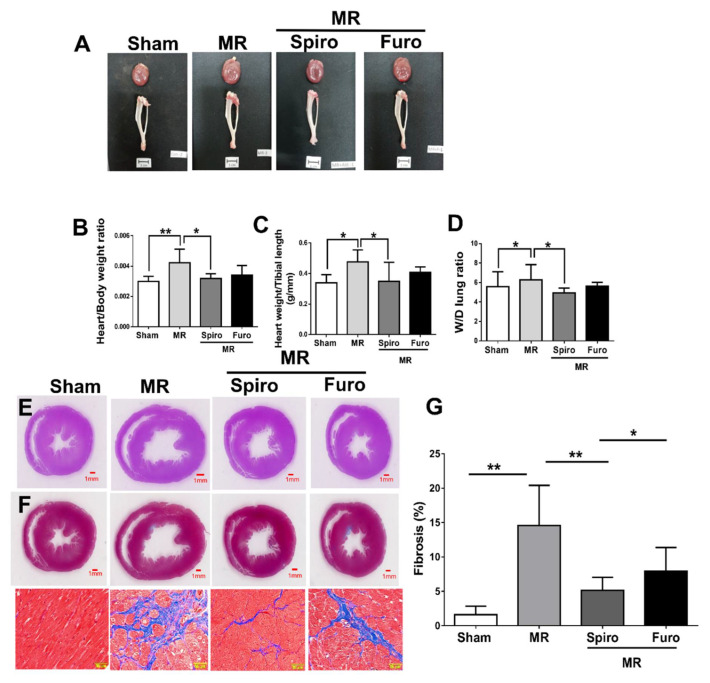
Effects of mineralocorticoid receptor antagonists on cardiac injury and fibrosis in rat model of mitral regurgitation (MR). (**A**) Images of harvested hearts and tibia in each group. (**B**) Representative heart weight/body weight, (**C**) heart weight/tibial length, (**D**) wet-to-dry lung weight ratio. (**E**) Images of heart sections with H&E staining and (**F**) Masson trichrome staining (blue) in the indicated groups; scale bars, 50 µm. (**G**) Quantification of cardiac fibrosis in the indicated groups of rats. Data are expressed as the mean ± standard deviation (S.D.). * *p* < 0.05 and ** *p* < 0.01 for differences from each group.

In histological analysis, the MR group had an increased heart-to-body-weight and heart-weight-to-tibial-length ratio, while Spiro—but not Furo—treatment attenuated volume-overload-triggered cardiomegaly (Figure 3A–C). Similarly, wet-to-dry (W/D) lung ratio was increased in the MR group but was attenuated in Spiro groups (Figure 3D). H&E and Masson trichrome staining were used to examine cardiac morphology and cardiac fibrosis in the sham, MR, MR+Spiro, and MR+Fluro groups, respectively (Figure 3E,F). Figure 3G revealed a significant increase in cardiac fibrosis in MR rat hearts compared with sham hearts. The level of cardiac fibrosis in the LV region was markedly attenuated by treatment with Spiro but not with Furo. Notably, the level of cardiac fibrosis detected in the Spiro treatment group remained significantly lower than that in the Furo treatment group.

### 3.4. Spiro Treatment Attenuated Cardiac Apoptosis by Mediating Mineralocorticoid Receptors (MCRs) in MR Rats

**Figure 4:**
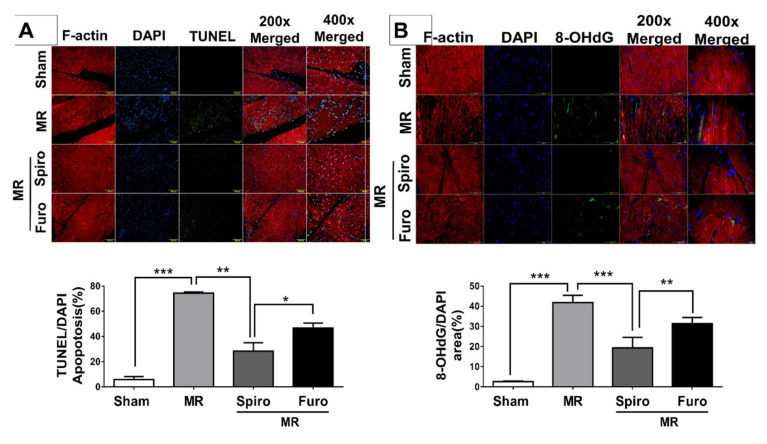
Mineralocorticoid receptor antagonists attenuate suppressed mitral regurgitation (MR)-induced cardiomyocyte apoptosis. (**A**) Representative heart sections with TUNEL staining for apoptosis detection (green) in 200× and 400× magnificence of scales; scale bars, 100 µm (top panel). Quantification of cardiac apoptosis in the indicated groups of rats (bottom panel). (**B**) Representative and quantified ROS expressions using hydroxydeoxyguanosine (8OHdG) staining. Data are expressed as the mean ± standard deviation (S.D.). * *p* < 0.05, ** *p* < 0.01, and *** *p* < 0.001 for differences from each group.

**Figure 5:**
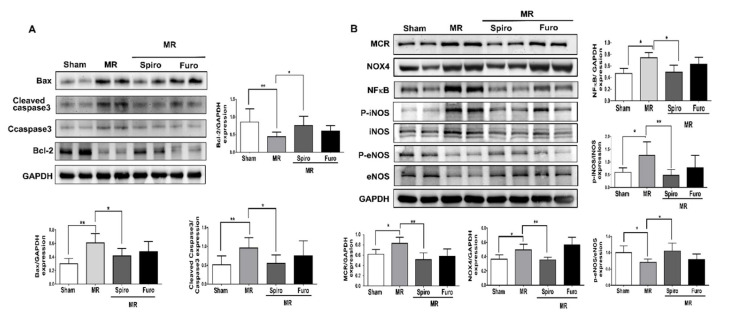
Mineralocorticoid receptor (MCR) antagonists attenuate suppressed mitral regurgitation (MR)-induced cardiomyocyte apoptosis associated with the MCR/NOX4/ROS pathway. (**A**) Representative and quantified apoptosis-associated proteins, including Bax, Bcl-2, and cleaved caspase 3, in the indicated groups of rats. (**B**) Representative quantification of ROS-associated proteins, including MCR/NOX4/NFκB/iNOS/eNOS, in the indicated groups of rats. Data are expressed as the mean ± standard deviation (S.D.). * *p* < 0.05 and ** *p* < 0.01 for differences from each group.

We also measured the level of apoptosis in cardiomyocytes using TUNEL staining. Compared to that of the sham group, the number of apoptotic cardiomyocytes significantly increased in the LV tissue of the MR groups, while pretreatment with Spiro significantly decreased the number of apoptotic cardiomyocytes and was even lower than that in the Furo treatment group (Figure 4A). Further, to measure the expression of ROS, using 8OHdG, a widely used biomarker of oxidative DNA damages, we observed a significant increase in ROS expression in the left ventricle tissue of MR rats, while Spiro significantly suppressed it but Furo treatment did not (Figure 4B). Apoptosis-related proteins, including Bax, cleaved caspase 3, and Bcl-2 in LV tissue were measured by Western blotting. As demonstrated in Figure 5A, compared with the sham groups, Bax and cleaved caspase 3 protein expression were upregulated, while Bcl-2 protein expression was downregulated in the MR groups. Notably, these changes in protein expression were reversed in the Spiro groups. MR-induced oxidative stress contributes to increased production of ROS, resulting in elevated cardiac apoptosis [7,11,12]. Recently, studies have demonstrated that NOX4 is an important source of ROS derived from mineralocorticoid receptors. To study the mechanisms involved in the cell protective effect of Spiro, we found that the protein levels of MCRs and NOX4, NADPH oxidases, were significantly increased in the MR groups, while Spiro treatment suppressed them (Figure 5B). Notably, Spiro also attenuated mitral regurgitation-induced activation of NFκB and iNOS, ROS-associated proteins, and in contrast, augmented eNOS signaling, which is associated with the suppression of apoptosis in cardiomyocytes (Figure 5B). Taken together, in addition to being a diuretic, Spiro improved MR-induced cardiovascular dysfunction. Additionally, the treatment of Spiro was associated with the mitigation of mitral regurgitation-triggered ROS and apoptosis-associated proteins.

### 3.5. Spiro Improved the Severity of MR in Patients

To verify our observation regarding the effects of Spiro on the mitigation of MR, using a clinical cohort, we aimed to test the clinical applications of Spiro in patients with degenerative MR. By analyzing the data from 213 and 252 patients with degenerative MR who received Spiro and Furo, respectively, we found that half the patients were male and had an average age of 60 years (Table 1). Approximately seventy percent of the patients had hypertension, diabetes, or chronic kidney disease, while a quarter of them had documented coronary artery disease or hyperlipidemia. Three-fourths of the patients were deemed New York functional class (NYFc) II. Approximately half of the patients were also on ACEIs/ARBs, while notably, the baseline clinical characteristics were similar between the Spiro and Furo groups.

With regard to the echocardiographic parameters, at baseline, the left atrial and ventricular chamber sizes and mass index were similar between the Spiro and Furo groups (Table 2). Left ventricular systolic function at baseline was preserved in both the Spiro and Furo groups (mean left ventricular ejection fraction; LVEFs 51.4 ± 10.1% vs. 52.5 ± 10.1%, *p* = 0.64). After six months of treatment, the LAVI was significantly reduced in patients treated with Spiro compared with those treated with Furo (changes in LAVI −6.62 ± 19.21 mL/m^2^ vs. −2.88 ± 17 mL/m^2^, *p* = 0.02). Moreover, Spiro users presented a trend of reduction in left ventricular systolic and diastolic volumes and an insignificant improvement in LVEF compared with patients treated with Furo (changes of LVEF 2.3 ± 11.8% vs. −0.5 ± 9.7%, *p* = 0.06). Notably, the severity of primary MR was observed to be similar between the two groups before treatment (that is, the mean EROA of MR 0.59 ± 0.11 cm^2^ vs. 0.62 ± 0.09 cm^2^, *p* = 0.39 and mean regurgitant volume 68.9 ± 17.9 mL vs. 67.4 ± 17.2 mL, *p* = 0.77). In terms of the severity of MR, the improvement in EROA was more significant in the group receiving Spiro than in the group receiving Furo (changes in EROA −0.23 ± 0.15 cm^2^ vs. −0.07 ± 0.11 cm^2^, *p* = 0.01). Similarly, the reduction in regurgitant volume was more significant in patients treated with Spiro than in those treated with Furo (changes in regurgitant volume −14.5 ± 23.2 mL vs. −9.4 ± 19.05 mL, *p* = 0.01).

## 4. Discussion

**Figure 6:**
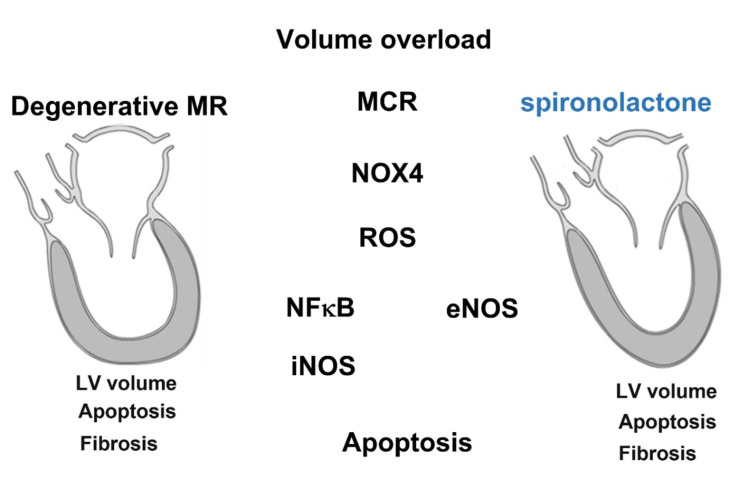
Summary of mineralocorticoid receptor (MCR) antagonists in mitigating mitral regurgitation (MR)-induced cardiac injury and apoptosis through the MCR/NOX4 pathway. Cardiomyocytes subjected to MR stress increase MCR and NOX4 expression and induce iNOS expression and inhibit eNOS expression, which contributes to apoptosis. This phenomenon could be prevented by inhibiting MCR expression with mineralocorticoid receptor antagonist treatment.

Degenerative MR is a highly prevalent heart disease that leads to significant rates of hospitalization and often requires cardiac surgery [2]. The mechanism of MR-induced HF is complex and is currently the focus of cardiovascular research. Although upon current concepts, cardiac fibrosis and remodeling are the major contributors to the pathology of MR induced HF, the detailed pathogenesis of MR-induced HF remains unclear. The mechanical induction of fibrotic remodeling and fibrosis, which are exposed to the influence of blood flow-induced shear stress, is a critical step leading to heart failure [13,14]. Moreover, volume overload-induced mechanical stress triggers ROS and inflammatory cytokine production [15]. Previous studies indicated that ROS were associated with myofibrillar degeneration and cardiac remodeling in patients with MR. Although surgical interventions have been used to arrest the pathophysiologic process and to delay cardiac remodeling, some patients fail to tolerate high-risk invasive procedures, and the postoperative recovery of left atrium function remains difficult to predict [16]. Most importantly, effective medical therapies to prevent MR-induced HF and delay the need for surgery are lacking. Our study, using multiple modalities and a dataset from bench to bedside, demonstrates some potential underlying physiological pathways, which should be taken into account when advocating certain therapies to patients, such as Spiro (Figure 6).

The MCRs, a pivotal regulator of blood pressure and renal sodium handling, has been found to promote vasoconstriction and fibrosis in the cardiovascular system. Previous studies have also shown that dysregulation of mineralocorticoid signaling is associated with hypertension, obesity, diabetes, and HF. Cezar et al. demonstrated that early Spiro use attenuated HF by improving cardiac remodeling in spontaneously hypertensive rats [17]. Similarly, Milliez et al. reported that Spiro reduced myocardial fibrosis in rats with myocardial infarction [18]. Conversely, Furo, a loop diuretic agent, contributed to a higher mortality in a rat model of chronic HF [19]. In contrast to Spiro, which blocks the renin–angiotensin–aldosterone system (RAAS) in patients with HF, Furo has been shown to trigger neurohumoral activity. Previous large-scale randomized studies, including RALES and EMPHASIS-HF, reported that the use of MRAs improve cardiac function by reduced ejection fraction results in decreasing the risk of both morbidity and death in patients with heart failure [7,20]. Likewise, in the EPHESUS trial, eplerenone, a second-generation mineralocorticoid receptor antagonist, lowered hospitalization for heart failure and mortality among patients with heart failure post-myocardial infarction [20]. In terms of cardiovascular death, the treatment with Spiro failed to show a significant result in the TOPCAT study, but it exhibited a significant reduction in HF hospitalization in patients having heart failure with preserved ejection fraction compared to those treated with the placebo [19,20]. Moreover, in EuroSMR, a European multicenter registry, including functional MR patients with left ventricular ejection fraction <50% indicated that triple-guideline-directed medical therapies, including MRAs, are associated with better survival after transcatheter edge-to-edge repair [21]. Therefore, the 2021 European Society of Cardiology as well as the 2022 American Heart Association/American College of Cardiology guidelines confirm the class I indication for mineralocorticoid receptor antagonist in patients with HF and a reduced left ventricular ejection fraction [22,23].

Although previous studies indicated that MRAs could ameliorate clinical outcome in patients with HF, whether MRAs are more effective in HF with different etiologies remains unknown [24]. Ibarrola et al. reported that mineralocorticoid receptor antagonist treatment reduced mitral valve thickness and proteoglycan content [5]. However, its feasibility in mitigating MR-induced myocardial dysfunction requires more investigation. In the present two-part study design, we showed that Spiro effectively attenuated MR-induced cardiovascular dysfunction in both clinical patents and preclinical animal models.

Overactivation of the MCRs has been observed in different cardiovascular diseases, including HF. The left ventricular dilatation, cardiac hypertrophy, and development of HF were attenuated, while mineralocorticoid receptor genes were inactivated in mouse models of chronic pressure overload or myocardial infarction (MI). Conversely, the use of Spiro has been found to reduce cardiac oxidative stress and nitrite generation in the setting of diabetes. Likewise, Kobayashi et al. reported that in Dahl salt-sensitive rats with HF, suppressing inducible NO synthase and nuclear factor kappa B (NF-κB), eplerenone stimulates eNOS and improves cardiac function and remodeling. To date, there is increasing evidence for a crucial role of aberrant mineralocorticoid receptor activation in HF with clinical studies showing the beneficial effects of mineralocorticoid receptor blockage. Ayuzawa et al. reported that treatment with eplerenone inhibited mineralocorticoid receptor signaling and Nox4 gene upregulation to improve cardiac hypertrophy and dysfunction caused by pressure overload [25]. Additionally, it has been demonstrated that aldosterone-induced Kv1.5 expression was suppressed by Spiro through attenuating MR-Nox1/2/4-mediated ROS generation in cardiomyocytes [14]. In this study, we also observed that Spiro attenuated MR-induced activation pathways of MCR/NOX4/ROS/iNOS, and in contrast, augmented eNOS signaling. There are some limitations of this study. First, given a correlative analysis, this study could not mechanistically provide the direct mechanism linking MRAs and myocardial dysfunction. In addition, the positive effect of Spiro and its high relevance for the treatment of heart failure over furosemide are well-known [21,22,26]. However, through this experimental model of MR we highlighted new insights of applications of heart failure drugs in mitigating the progression of MR and the possibilities of clinical interest for further studies. Further interventional studies focusing on the MCR/NOX4-associated pathway are necessary. Second, in this two-part study combining a prospective, experimental arm and the other retrospective, clinical arm, we aimed to seek the potentials of clinical applications of Spiro in patients with degenerative MR. Although the main results could be discussed separately, it provides a translational approach regarding the role of MRAs in MR. Most importantly, instead of an observational study, a randomized control study to investigate the effects of ARNI on degenerative MR is crucial in the future. Lastly, although hyperkalemia remains a concern with mineralocorticoid receptor antagonists, third-generation mineralocorticoid receptor antagonists such as finerenone may present a more favorable cardiac-to-renal activity ratio and fewer adverse effects. The ongoing FINEARTS-HF study should provide evidence regarding the effects of finerenone on outcomes in patients with HF. In this study, we addressed evidence in terms of the potential benefits of Spiro use in mitigating mitral regurgitation-induced myocardial fibrosis and dysfunction. Our findings provide alternative options for pharmacological intervention in treating patients with mitral regurgitation-induced HF.

## 5. Conclusions

Collectively, our findings demonstrate that MR-induced cardiovascular dysfunction was effectively attenuated by Spiro treatment as compared with Furo treatment both in clinical and preclinical studies. The potential indication of Spiro use in slowing the progression of degenerative MR and its induced HF requires further investigation, especially that of randomized controlled multicenter clinical trials.

## Figures and Tables

**Table 1:** The baseline characteristics of patients with moderate/severe mitral regurgitation receiving either spironolactone or furosemide (N = 465).

Parameters	Spironolactone (N = 213)	Furosemide (N = 252)	*p* Value
Age (y/o)	61.8 ± 14.8	61.1 ± 16.5	
Male gender, n (%)	109 (51.1)	117 (46.4)	0.35
BMI (kg/m^2^)	22.9 ± 5.08	23.3 ± 4.6	0.24
Heart rate (bpm)	80 ± 17.2	80.1 ± 17.5	0.78
SBP (mmHg)	159.6 ± 30.6	160.2 ± 32.5	0.36
DBP (mmHg)	76.5 ± 16.4	78.9 ± 17.3	0.43
Hypertension, n (%)	153 (71.8)	178 (70.6)	0.42
Diabetes, n (%)	148 (69.4)	182 (72.2)	0.29
CAD, n (%)	53 (24.8)	63 (25)	0.25
HF NYFcII, n (%)	161 (75.5)	205 (81.3)	0.28
HF NYFcIII, n (%)	52 (24.4)	47 (18.6)	0.15
Hyperlipidemia, n (%)	49 (23)	58 (23)	0.74
CKD (including H/D), n (%)	61 (28.6)	59 (23.4)	0.12
eGFR (mL/min/1.73m^2^)	51.1 ± 30.5	55.1 ± 30.9	0.83
ALT(mg/dL)	24.1 ± 33.8	28.1 ± 44.9	0.53
Total cholesterol(mg/dL)	149.3 ± 42.7	146.8 ± 41.3	0.45
LDL(mg/dL)	83.7 ± 33.28926	81.1 ± 34.4	0.25
NT-proBNP (pg/mL)	331.1 ± 756.1	299.3 ± 465.2	0.29
Concomitant medications			
ACEIs/ARBs	102 (47.8)	118 (46.8)	0.42
β-Blocker	45 (21.1)	63 (25)	0.34
Statin	34 (15.9)	48 (19)	0.63
Antiplatelet/anticoagulants	63 (29.6)	76 (30.1)	0.28

Data are expressed as mean ± SD or number (%). *p* < 0.05 as significance. BMI = body mass index; SBP = systolic blood pressure; DBP = diastolic blood pressure; CAD = coronary artery disease; HF = heart failure; AF = atrial fibrillation, paroxysmal or persistent; CKD = chronic kidney disease; eGFR = estimated glomerular filtration rate; ALT = alanine aminotransferase; LDL = low-density lipoprotein; NT-proBNP = NT-pro B-type natriuretic peptide; ACEIs/ARBs = angiotensin-converting enzyme inhibitors/angiotensin receptor blockers; medications = continuous prescription for more than three months.

**Table 2:** The echocardiographic characteristics of patients with moderate/severe mitral regurgitation receiving either spironolactone or furosemide (N = 465).

Parameters	Spironolactone (N = 213)	Furosemide (N = 252)	*p* Value
Echocardiographic Characteristics			
Left atrial volume index (mL/m^2^)			
Baseline	51.7 ± 20.67	50.7 ± 19.74	0.6
Follow-up	45.1 ± 15.87	47.9 ± 16.63	0.06
Changes	−6.62 ± 19.21	−2.88 ± 17	0.02
LVESV, mL			
Baseline	39.8 ± 12.86	40 ± 16.84	0.9
Follow-up	35.7 ± 13.5	37.8 ± 13.9	0.11
Changes	−4.1 ± 11.4	−2.2 ± 10.3	0.06
LVEDV, mL			
Baseline	89.2 ± 23.3	91.9 ± 24.73	0.13
Follow-up	76.9 ± 22.2	83.5 ± 21.5	0.01
Changes	−12.3 ± 22.12	−8.4 ± 21.06	0.05
LVEF (%)			
Baseline	51.4 ± 10.1	52.5 ± 10.1	0.64
Follow-up	53.7 ± 9.3	51.9 ± 9.9	0.21
Changes	2.3 ± 11.8	−0.5 ± 9.7	0.06
LV mass index (g/m^2^)			
Baseline	117.5 ± 34.2	115.3 ± 38.5	0.95
Follow-up	107.1 ± 34.8	107.2 ± 28.3	0.4
Changes	−10.4 ± 35.1	−8.1 ± 31.5	0.12
EROA of MR (cm^2^)			
Baseline	0.62 ± 0.09	0.57 ± 0.1	0.06
Follow-up	0.39 ± 0.14	0.5 ± 0.11	0.01
Changes	−0.23 ± 0.15	−0.07 ± 0.11	0.01
Vena contracta (cm)			
Baseline	1.02 ± 0.47	0.94 ± 0.43	0.07
Follow-up	1.04 ± 0.37	0.95 ± 0.36	0.13
Changes	−0.01 ± 0.4	0.01 ± 0.45	0.5
Regurgitation volume (mL)			
Baseline	70.6 ± 19.8	64.5 ± 16.03	0.1
Follow-up	56.1 ± 20.5	55.0 ± 17.8	0.5
Changes	−14.5 ± 23.2	−9.4 ± 19.05	0.01

LVESVI = left ventricular end-systolic volume index; LVEDVI = left ventricular end-diastolic volume index; LVEF = left ventricular ejection fraction; EROA = effective regurgitant orifice area; MR = mitral regurgitation.

## Data Availability

The original data is available upon reasonable requests to the corresponding author.

## Supplementary Materials

The following supporting information can be downloaded at: https://www.mdpi.com/article/10.3390/cells11172750/s1, Figure S1: Study design of mineralocorticoid receptor antagonists on cardiac remodeling in a rat model of mitral regurgitation (MR); Supplemental methods.
