# Supplementary material for: Mineralocorticoid Receptor Antagonists Mitigate Mitral Regurgitation-Induced Myocardial Dysfunction
Source: Cells. 2022 Sep 3;11(17):2750. doi: 10.3390/cells11172750 (PMC9455158; doi:10.3390/cells11172750)
Supplement: Supplementary file 1 [file cells-11-02750-s001.zip › Figure S1.pdf]

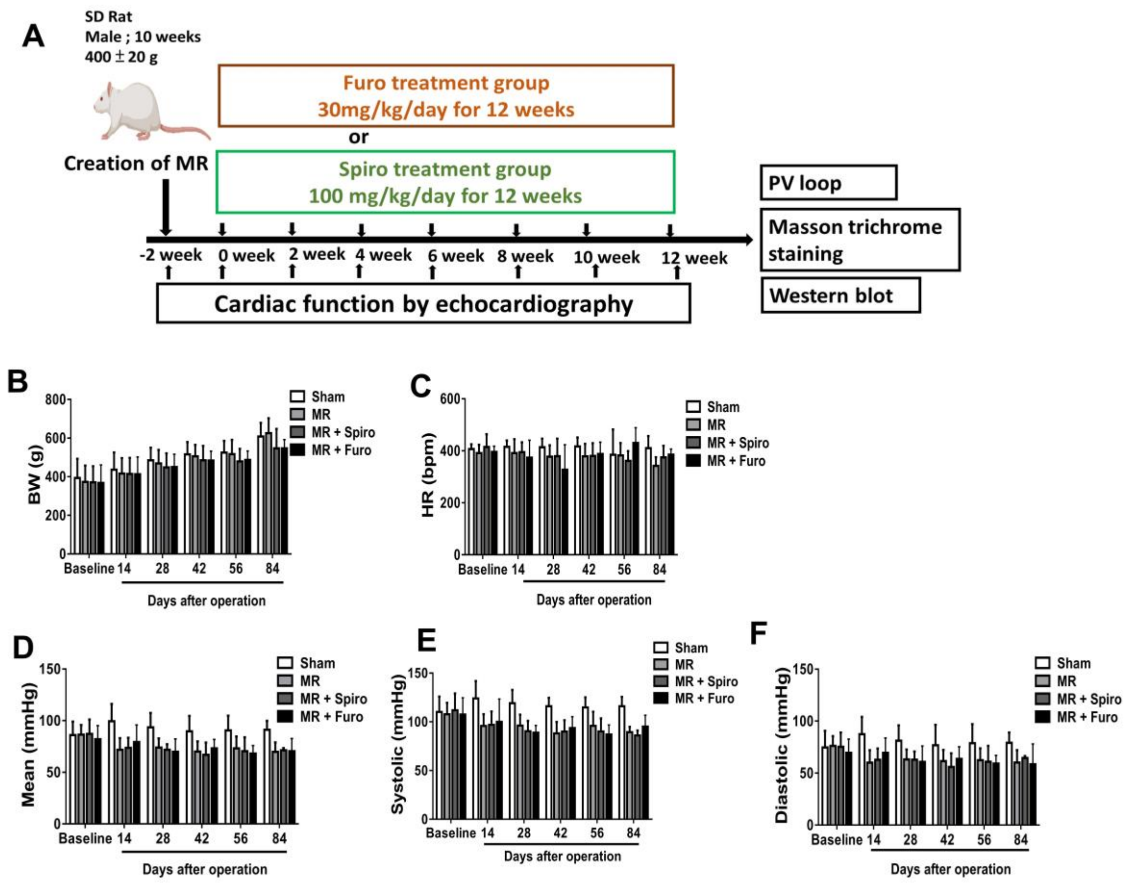

Figure S1. Study design of mineralocorticoid receptor antagonists on cardiac remodeling in a rat model of mitral regurgitation (MR).
