# Supplementary material for: Mineralocorticoid Receptor Antagonists Mitigate Mitral Regurgitation-Induced Myocardial Dysfunction
Source: Cells. 2022 Sep 3;11(17):2750. doi: 10.3390/cells11172750 (PMC9455158; doi:10.3390/cells11172750)
Supplement: Supplementary file 1 [file cells-11-02750-s001.zip › Supplemental methods.pdf]

Supplemental methods

Echocardiographic parameters in patients

The chamber dimensions including left ventricular end-diastolic volume index (LVEDVI), left ventricular end-systolic volume index (LVESVI), and left ventricular mass index (LVMI) were measured using the two-dimensionally guided M-mode method[9]. Left ventricular ejection fraction (LVEF), was measured by both apical 2- and 4- chamber Simpson method. Left atrial volume index (LAVi) was measured based on the maximal LA area in both apical 2- and 4- chamber views and the length from the posterior wall to the MV hinge, divided by body surface area. EROA was determined by dividing the regurgitant flow rate, calculated as $2\pi r2\times$ aliasing velocity, where r is the proximal isovelocity surface area (PISA) radius, by peak MR velocity[8]. A regurgitant volume was estimated as EROA multiplied by the velocity time integral of the MR jet. The vena contracta (VC), a large MR jet with a large proximal flow convergence region was measured in late systole while the width of VC is seen corresponding to the severity of MR.

Echocardiographic and strain analyses in animals

Doppler, two-dimensional (2-D), and 2-D guided M-mode images were recorded from parasternal long-axis and parasternal short-axis and apical four-chamber views. Echocardiograph measurements included VC, chamber sizes, interventricular septal thickness at end-diastole (IVSd), left ventricular internal dimension at end-diastole (LVIDd), fractional shortening (FS). For strain analysis, with frame rates around 150 frames/s the endocardial border of left ventricle (LV) at the parasternal long axis view was tracked and followed by defining the width of myocardium using EchoPAC software. Echocardiography including global longitudinal strain (GLS) was performed at baseline and weekly during the duration of study.

Hemodynamic Study of Pressure-Volume Loop (PV loop)

After rats were anesthetized, the chest of the rat was opened through a median sternotomy and created a fine hole in the apex area of the LV using a 27-gauge needle. A Millar pressure catheter (SPR-838; Millar Instruments, Houston, TX, USA) was gently introduced into the LV in order to avoid contact with the LV and septal walls, which was ensured by observing the PV loop formation. The jugular vein was cannulated for infusion of 10% hypertonic saline to determine parallel conductance. The inferior vena cava (IVC) was exposed for the occlusion of inferior vena cava using a 3-0 surgical silk. The hemodynamics of LV were recorded using the

PowerLab converter (Millar Instruments) and were analyzed using LabChart software (ADI Instruments). End-systolic (Ves), end-systolic (Pes), the maximal velocity of pressure rise (+ dP/dt), fall (− dP/dt), and arterial elastance (Ea) were recorded to evaluate LV systolic function. The end-diastolic volume (Ved), end-diastolic pressure (Ped), and the time constant of isovolumic pressure decay (tau) were recorded to evaluate LV diastolic function. Additionally, the end-systolic pressure-volume relationship (ESPVR) and end-diastolic pressure-volume relationship (EDPVR) was measured by transient occlusion of the inferior vena cava.

Western Blot

LV tissues from each group were collected and homogenized in a lysis buffer, which contained 20 mM Trisbase (pH 8.0), 150 mM NaCl, 1.0% Nonidet P-40, and protease inhibitors (Roche, Basel, Switzerland). The homogenates were sonicated and centrifuged at 12,000 x g for 30 min at 4˚C. The protein concentration was determined using a bicinchoninic acid protein assay kit (BCA; Thermo Fisher Scientific) according to the manufacturer's protocol. The total protein (20 μg) was separated by sodium dodecyl sulfate-polyacrylamide gel electrophoresis (SDS-PAGE), and transferred electrophoretically to polyvinylidene fluoride (PVDF) membranes (MERCK Millipore, Massachusetts, USA). After washing, the membrane was blocked in 5% nonfat milk for 2 hours at room temperature and then was incubated at 4˚C overnight with the corresponding primary antibody, including mineralocorticoid hormone receptor (MCR; 1:2000 Santa Cruz, Biotechnology; Dallas, TX, USA), NADPH Oxidase 4 (NOX4; 1:1000, Affinity Biosciences), nuclear factor kappa-B (NFκB; 1:1000, Abcam, Cambridge, UK), inducible nitric oxide synthase (iNOS) (1:1000, Proteintech, Rosemont, USA) and its phosphorylation form (p-iNOS; 1:1000, Invitrogen, Thermo Fisher Scientific, Massachusetts, USA), endothelial nitric oxide synthase (eNOS; 1:1000, Proteintech, Rosemont, USA) and its phosphorylation form (p-eNOS, 1:1000, Cell Signaling, Massachusetts, USA), B-cell lymphoma-2 (Bcl-2; 1:1000, Arigo Hsinchu,Taiwan, ROC), Bcl2 Associated X, Apoptosis Regulator (Bax; 1:1000), cleaved caspase 3 (1:1000, Cell Signaling, Massachusetts, USA), or glyceraldehyde-3-phosphate dehydrogenase (GAPDH; 1:5000, Sigma-Aldrich Co., St Louis, MO, USA). After washing, all membranes were incubated for 1 hour with the corresponding horseradish peroxidase (HRP) linked anti rabbit/mouse IgG secondary antibody. The membranes were analyzed by ECL-Western blotting system (AVEGENE CHEMX 400). The intensity of the protein band was normalized to the expression of GAPDH and quantification analyzed with Image J software (Bethesda, NIH, USA).
